# Analysis of the Imaging Characteristics of Holographic Waveguides Recorded in Photopolymers

**DOI:** 10.3390/polym12071485

**Published:** 2020-07-03

**Authors:** Cristian Neipp, Soumia Imane Taleb, Jorge Francés, Roberto Fernández, Daniel Puerto, Eva María Calzado, Sergi Gallego, Augusto Beléndez

**Affiliations:** 1 I.U. Física Aplicada a las Ciencias y las Tecnologías, Universidad de Alicante, P.O. Box 99, E-03080 Alicante, Spain; yousfi051291@gmail.com (S.I.T.); jfmonllor@ua.es (J.F.); roberto.fernandez@ua.es (R.F.); dan.puerto@ua.es (D.P.); evace@ua.es (E.M.C.); sergi.gallego@ua.es (S.G.); a.belendez@ua.es (A.B.); 2 Department de Física, Ing. de Sistemas y Teoría de la Señal, Universidad de Alicante, P.O. Box 99, E-03080 Alicante, Spain

**Keywords:** photopolymers, diffraction gratings, holographic waveguide, holography

## Abstract

In this work, we study the imaging characteristics of an optical see-through display based on a holographic waveguide. To fabricate this device, two transmission holograms are recorded on a photopolymer material attached to a glass substrate. The role of the holograms is to couple the incident light between air and the glass substrate, accomplishing total internal reflection. The role of noise reflection gratings and shrinkage on the imaging characteristics of the device will be also explored. The holograms (slanted transmission gratings with a spatial frequency of 1690 lines/mm) were recorded on a polyvinyl alcohol acrylamide holographic polymer dispersed liquid crystal (HPDLC) material. We will show that sufficient refractive index modulation is achieved in the material, in order to obtain high diffraction efficiencies. We will demonstrate that the final device acts as an image formation system.

## 1. Introduction

Optical waveguides play an important role in the construction of integrated optical circuits, and are essential in optical telecommunication applications [1]. The general optical waveguides used for these applications are the so called planar waveguides that consist of three layers of materials with different dielectric constants, allowing for total internal reflection in the second medium. Most waveguide devices are formed by diffusion of an impurity into glass or LiNbO3. Other interesting method to produce optical waveguides is by using the photorefractive effect in transparent materials, when exposed to an unfocused laser beam with enough intensity to initiate photorefractive effect, the self-focusing of the light beams record waveguides on the material [2]. By the same principle, self-written waveguides (SWW) [3] have also been fabricated in photopolymers, but, in this case, the mechanism under waveguide formation is photopolymerization.

Other important application of optical waveguides is in the fabrication of traditional head up displays and helmet mounted displays. In this case, the optical waveguide is fabricated by using holographic technologies [4]. The complex system of lenses used to generate a virtual image for the user is substituted by a holographic waveguide what reduces the size and weight of the display systems [5,6]. Based on the same principles, the holographic waveguides have also been introduced in the fabrication of “see through” glasses, which exploit the concept of augmented reality or virtual reality. The “glasses” have some advantages in relation to a mobile direct-viewing screen, for instance hands-free and high privacy characteristics.

A method to fabricate a holographic waveguide is by recording two holograms in a glass substrate: the first hologram acting as an in-coupler element, and the second hologram acting as an out-coupler element. Light is guided through the glass substrate by total internal reflection. In general, the couple in and couple out holograms were recorded as reflection gratings [7,8,9], but in recent works this technology has been expanded to work with transmission holograms as well [10,11]. A suitable medium for the recording of the holograms is a dry photopolymer material, since this material is a holographic recording media with important characteristics, such as low price, self-processing capability and a high versatility. Photopolymer materials have long been used as holographic recording material. There is great knowledge of its behavior, and they are useful in a great number of applications, such as diffractive optics [12,13], optical communications [14], photonic crystal [15] or sensors [16], among others. The process of hologram formation has also been greatly studied, and this is basically due to a competition between monomer diffusion and polymerization [17,18,19,20], although other ways of inscription of holographic information in photopolymer materials have been reported [21,22,23]. For instance, by using the theory of photoisomerization induced vectorial motion of matter, Moujdi et al. recorded surface relief gratings in azo-polymers [24]. The use of azo dye doped polymer films allowed also photomanipulation of the light diffracted by holographic gratings recorded on this kind of material [25].

For this particular application, the holographic photopolymer material should be adequately designed and optimized attending to two important requirements. Firstly, the material must be able to create sufficient index modulation for spatial frequencies higher that 1600 lines/mm [10], as will be explained in Section 2. Secondly, the shrinkage after the recording process must be controlled. This is because for this particular application slanted gratings are recorded and, as will be explained in Section 4, changes in thickness alter the geometry of the gratings and therefore the reconstruction conditions [26]. 

Another important fact that must be taking into account in the recording of the holographic waveguide is the possible recording of noise reflection gratings. In general, noise gratings in other devices extract a small amount of energy from the diffracted order, with no other spurious effect [27,28]. Nonetheless, in this particular device it will be shown that noise reflection gratings could create an unwanted secondary image. In Section 3, the different noise gratings recorded in the material are explained. Since the reconstruction is made with a different wavelength of that of the recording, the reflection noise gratings could be or not “visible” during reconstruction. The role of shrinkage or swelling in the “visibility” of these gratings is explained in Section 4.

In Section 5, the experimental setup and the composition of the photopolymer material will be presented. As a result of the comparison made in other works [10,11] among different polymer compositions, we used a holographic polymer dispersed liquid (HPDLC) photopolymer as the recording medium for the transmission diffraction gratings. In order to understand the capabilities of this material for this particular application, slanted transmission holographic gratings with spatial frequencies of 1690 lines/mm were recorded and analyzed. Finally, in Section 6, the image characteristics of the holographic waveguide will be evaluated. 

## 2. Theoretical Design of the Waveguide

**Figure 1:**
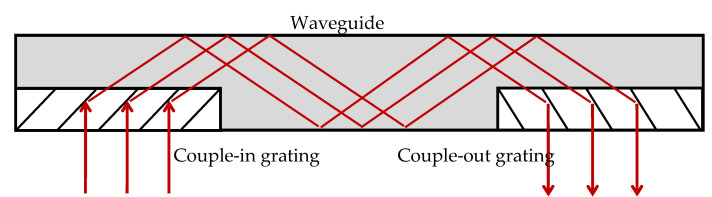
Holographic waveguide by two transmission holograms.

In this section, we will explain the design and reconstruction parameters of a transmission holographic waveguide. We will use the configuration of Figure 1. The first hologram in front of the lens couples the light to the waveguide in total reflection condition, while the second hologram couples the beam out of the waveguide. 

**Figure 2:**
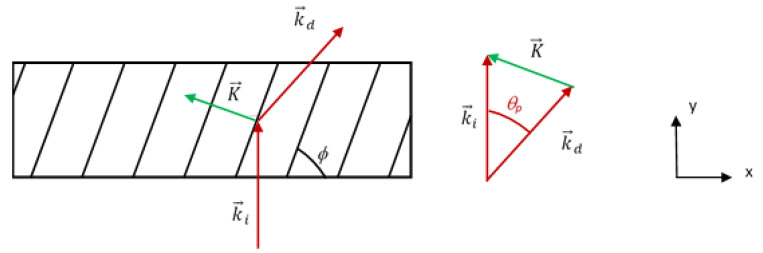
Couple-in diffraction grating.

The angular condition of total internal reflection is θ_p_ = 42.15° for the case of the photopolymer with refractive index of *n*_p_ = 1.49, and θ_g_ = 41.47° for the glass substrate, assuming a refractive index of *n*_g_ = 1.51. Assuming normal incidence it is possible to design the gratings parameters to accomplish the redirection of light in the way prescribed in Figure 1. Figure 2 shows the configuration of the couple-in grating, where it can be seen that the grating vector $\vec{K}$ redirects the incident to the diffracted ray, which verifies total internal reflection, that is this ray forms and angle of 42.15° with the y axis inside the grating. 

The grating vector $\vec{K}$ can be calculated as:

(1) $$\vec{K}={\vec{k}}_{i}-{\vec{k}}_{d}$$

where ${\vec{k}}_{i}$ is the propagation vector of the incident ray (in this case perpendicular to the interface air-grating) and ${\vec{k}}_{d}$ is the propagation vector of the diffracted ray. The moduli of them can be calculated as:

(2) $$\beta_{c}=\left|{\vec{k}}_{i}\right|=\left|{\vec{k}}_{d}\right|=\frac{2\pi }{\lambda_{c}}n_{p}$$

where *β*_c_ and *λ*_c_ are the moduli of the propagation vectors and the wavelength at the reconstruction step.

Two are the necessary design parameters:

(1) The period of the fringes, or the spatial frequency of the grating, that are related with the grating vector as follows:
  (3) $$\left|\vec{K}\right|=2\pi f=\frac{2\pi }{\Lambda }$$
(2) The angle *ϕ* formed by the interference fringes with the substrate that can be calculated by using:
  (4) $$\phi =\frac{\pi }{2}-\left|\mathrm{atan}\left(\frac{K_{y}}{K_{x}}\right)\right|$$

The couple out grating is designed in a similar manner, but in this case, the *K* vector is the reflection image of that of the couple in grating with respect to the y axis of Figure 2.

**Figure 3:**
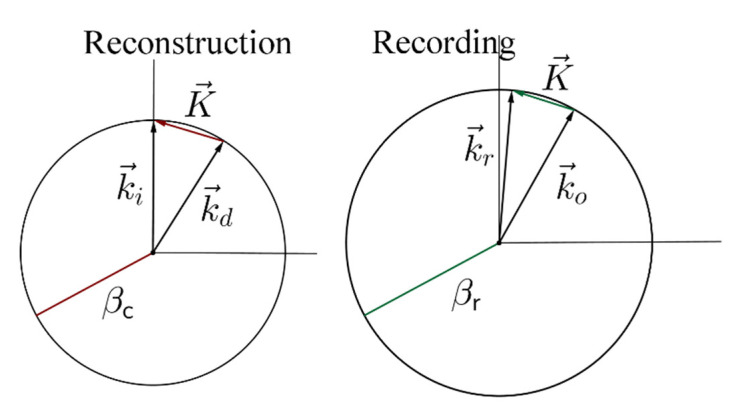
Recording and reconstruction geometry.

Once the design parameters of the gratings have been designed to accomplish total internal reflection, the gratings must be recorded. It is clear that these grating cannot be recorded using the wavelength of reconstruction, since the angle of the object beam should be π/2 in air. Therefore, the grating should be fabricated with a shorter wavelength. Figure 3 illustrates the configuration of reconstruction and recording. The propagation vectors ${\vec{k}}_{r}$, for reference wave, and ${\vec{k}}_{o}$, for object wave) in the recording process used to obtain the same $\vec{K}$ vector as that of the reconstruction step, but with a different wavelength, are different to those of the recording process. If, *β*_r_, is the modulus of these vectors, they are obtained as:

(5) $$\beta_{r}=\left|{\vec{k}}_{r}\right|=\left|{\vec{k}}_{o}\right|=\frac{2\pi }{\lambda_{r}}n_{p}$$

where *λ*_r_ is the recording wavelength and *n_p_* is the refractive index of the photopolymer.

It is possible to obtain the propagation vectors, ${\vec{k}}_{r}\;$ and ${\vec{k}}_{o}$, for prescribed values of $\vec{K}$ and *β*_r_ (the radius of the Ewald sphere) by using the following geometrical arguments:

Since ${\vec{k}}_{r}$ and ${\vec{k}}_{o}$ have the same modulus, the vectors (${\vec{k}}_{r}-{\vec{k}}_{o}$ = $\vec{K}$) and (${\vec{k}}_{r}+{\vec{k}}_{o}$) are perpendicular, so the dot product among them is zero:

(6) $$\vec{K}\cdot \left({\vec{k}}_{r}+{\vec{k}}_{o}\right)=0$$

On the other hand:

(7) $$\vec{K}\cdot \vec{K}=\vec{K}\cdot \left({\vec{k}}_{r}-{\vec{k}}_{o}\right)={\left|\vec{K}\right|}^{2}$$

From the two previous expressions the following equation can be obtained:

(8) $${\left|\vec{K}\right|}^{2}=2\vec{K}\cdot {\vec{k}}_{r}=-2\vec{K}\cdot {\vec{k}}_{o}$$

Therefore the angles of ${\vec{k}}_{r}$ and ${\vec{k}}_{o}$ with $\vec{K}\;$ are determined by

(9) $$\cos\left(\theta_{\hat{\vec{K}\;{\vec{k}}_{r}}\;}\right)=\frac{\left|\vec{K}\right|}{2\left|{\vec{k}}_{r}\right|}$$

And

(10) $$\cos\left(\theta_{\hat{\vec{K}\;{\vec{k}}_{o}}\;}\right)=-\frac{\left|\vec{K}\right|}{2\left|{\vec{k}}_{o}\right|}$$

Since the modulus of ${\vec{k}}_{r}$ and ${\vec{k}}_{o}$ are prescribed (*β*_r_), these vectors are completely determined if the modulus and angle of the grating vector are known.

In this work, the gratings were recorded with green light, λ_r_ = 532 nm and reconstructed with red light λ_c_ = 633 nm. For the recording, the angles of the reference and object propagation vectors with respect to the normal were: in air, θ_r_ = 5.2° and θ_o_ = 68.5°; and in the photopolymer, θ_r_ = 3.42° and θ_o_ = 38°.

## 3. Noise Gratings

**Figure 4:**
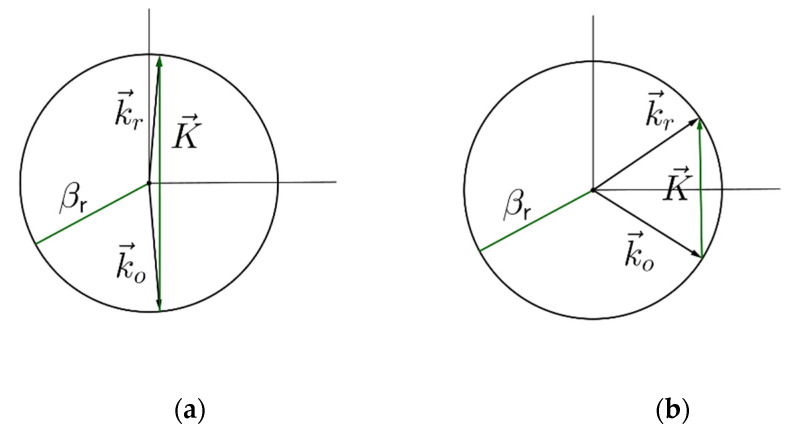

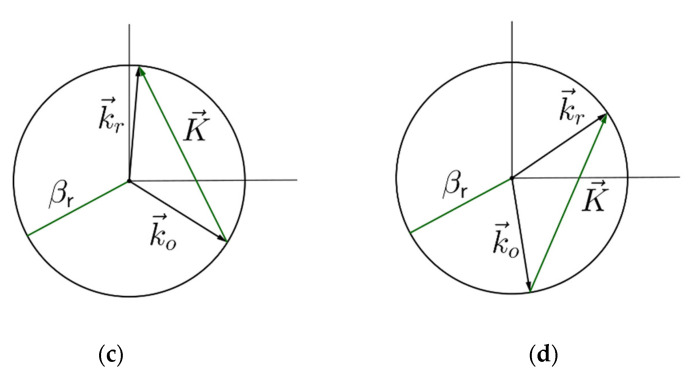
Noise gratings recorded in the photopolymer. (**a**) Noise grating due to the reference wave and its reflected counterpart. (**b**) Noise grating due to the object wave and its reflected counterpart. (**c**) Noise grating due to the reference wave and the reflected object wave. (**d**) Noise grating due to the object wave and the reflected reference wave.

During the recording process of diffraction gratings in photosensitive materials, the self-interference of the incident beam with scattered radiation or reflected beams produce gratings, the effect of which, at reconstruction, is to bring about a reduction in diffraction efficiency and signal-to-noise ratio [27]. In the case of the use of photographic emulsions as recording materials, the fine-grained silver halide crystals suspended in gelatin act as scatterers producing noise gratings, due to scattering [28]. Although, in photopolymers, this effect is less important, noise gratings due to scattering have also been studied in this kind of material. The other types of noise gratings that usually appear are due to the interference of the reference and object beam with the beams reflected in the interface glass-air. Figure 4 shows the four different noise reflection gratings that are recorded in the volume hologram recorded with the designed parameters explained in Section 2. These gratings are produced due to the interference of the incident beams with the reflected ones. For instance, the reflected grating produced in Figure 4c is due to the interference of the reference beam (of Figure 3) with the reflected object beam (of Figure 3). 

Although these are reflection gratings created during the recording process they could be or not “visible” by the reconstruction beam in the reconstruction process. To explain this fact, we should look at Equations (9) and (10), which give in fact the two Bragg angular conditions. Since the cosine is bounded by [−1, 1] the reflection gratings recorded with the recording wavelength will be “visible” by the reconstruction beam if the modulus of the K vector is less than two times the modulus of the propagation vector in the reconstruction process (*β*_c_). That is:

(11) $$\frac{K}{2\beta_{c}}\leq 1$$

For instance, with the recording and reconstruction conditions explained in Section 2 the *K* vector of Figure 4d has a modulus of 32.8 μm^−1^ and forms an angle of 17.6° with the y axis. The value of K/2*β*_c_ is 1.11 so this grating is not reconstructed with red light. As we will see in the next section, things change when either swelling or shrinkage is present in the hologram. It can be demonstrated that in the absence of changes in the thickness of the grating only the noise grating of Figure 4b can be reconstructed with red light.

## 4. Changes in the Thickness of the Grating 

During and after recording, there exist variations of the thickness of the material. In photographic emulsions these changes are produced in the chemical procedure, for instance, when the plates are bleached by rehalogenating baths. The substitution of metallic silver by silver halide grains produce shrinkage due to the lower size of the silver halide grains. In photopolymers, the variation of thickness is produced during the polymerization process, and is usually associated with shrinkage. Other factors can also influence the variations of thickness in volume holograms after the recording process, such as the humidity conditions, the processes for stabilizing and conserving the holograms, etc. 

**Figure 5:**
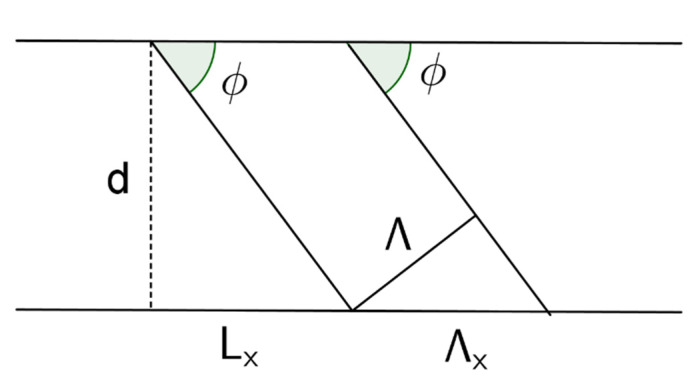
Geometry of the interference fringes.

The variation of thickness after the gratings have been recorded, alter the geometrical parameters of the gratings. There are different models that explain how a variation of thickness influence on the gratings. Here, we will briefly expose the ideas of [26], to relate shrinkage with the change in Bragg condition of the grating. It will be assumed that one side of the material is attached to a rigid substrate, which is the basis of the fringe rotation model [29]. In Figure 5, *ϕ* is the angle made by the fringes with the x axis, d is the thickness of the grating, Λ, is the period of the grating, Λ_x_ is the period along the rigid substrate, and L_x_ is the projection of the length of a fringe onto the x axis. 

The relations among these parameters are easily calculated by geometrical arguments:

(12) $$\Lambda_{x}=\frac{\Lambda }{\sin\phi }$$

(13) $$L_{x}=\frac{d}{\tan\phi }$$

In this model, it is assumed that Λ_x_ and L_x_ are constant during the shrinkage or swelling process. That is, that the ends of the fringes attached to the substrate do not move during the process. Therefore, if Λ’, *ϕ*’ and d’ denote the new period, angle, and thickness after a variation of thickness process, one can obtain the following relations:

(14) $$\Lambda ’=\frac{\Lambda \sin\phi ’}{\sin\phi }$$

(15) $$d’=\frac{d\tan\phi ’}{\tan\phi }$$

From Equation (15), if one knows the shrinkage or swelling factor, it is possible to determine the new angle made by the fringes with the substrate. On the other hand, once this new angle is known, one can obtain the period of the grating and therefore the modulus of the new *K* vector by using Equation (14). 

If one assumes small changes of thickness, differencing Equations (14) and (15) in combination with Equations (12) and (13), the following relation is obtained [26]:

(16) $$\frac{\Delta \Lambda }{\Lambda }=\cos^{2}\phi \frac{\Delta d}{d}$$

where ΔΛ is the variation of the period and Δd the variation of the thickness.

Some interesting conclusions can be obtained if one takes into account Equation (16), and the discussion of Section 2 and Section 3. The sign of the change in the period is the same as that of the variation of the thickness. Considering the relation among the period and the modulus of the *K* vector, this means that shrinkage increases the modulus of the *K* vector, while swelling decreases it. Therefore if condition (11) is violated shrinkage increases the inequality. In the particular case of the noise gratings of Figure 4a,c,d, the presence of some of them will be an indication of swelling. Moreover, it is possible to obtain the swelling or shrinkage factor needed to observe one of these gratings in the reconstruction process. Differencing the *K* vector in terms of the period and using Equation (16) one can obtain:

(17) $$\frac{\Delta K}{K}=-\cos^{2}\phi \frac{\Delta d}{d}$$

If we denote *K’* as the grating vector after shrinkage or swelling, *K’* takes the value of 2*β*_c_ in the limiting condition of Equation (11). Now, using Equation (17), the limiting swelling factor in order for the noise reflections gratings to be present in the reconstruction process can be calculated as:

(18) $$\frac{\Delta d}{d}=\frac{1}{\cos^{2}\phi }\left(1-\frac{2\beta_{c}}{K}\right)$$

**Figure 6:**
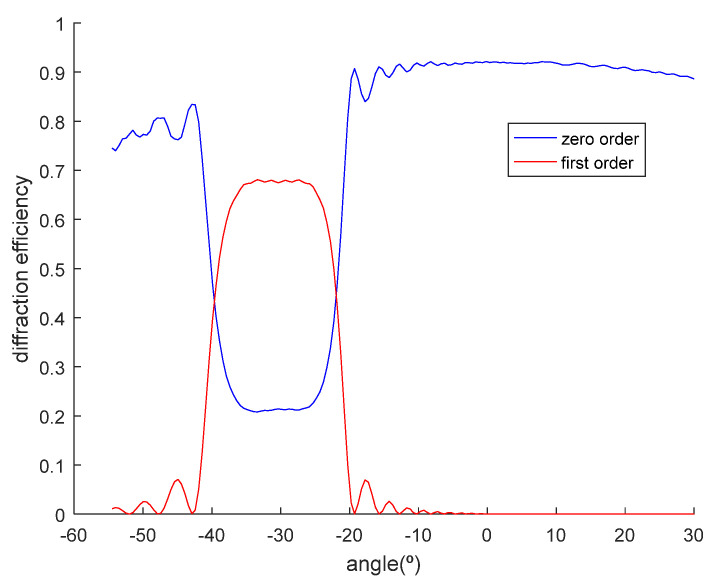
Diffraction and transmission efficiency as a function of the angle for a noise reflection grating after swelling.

For the particular case of grating of Figure 4d, the swelling factor defined as d’/d must be of 1.12, in order for that grating to be “visible” in the recording process. The Bragg angles obtained by conditions (9) and (10) in air (after applying Snell’s law) are 32.8° and 27.26°. Whereas this grating will not be “visible” without swelling, the changes in the *K* vector after swelling permit its reconstruction. In Figure 6, a simulation made by using the rigorous coupled-wave theory [30] is shown, for the reflection grating of Figure 4d after swelling with a refractive index modulation Δ*n* = 0.0085 and an initial thickness of d = 30 μm.

## 5. Experimental Setup

Attending to the photopolymer optimization developed in a previous work [11], and the studies of the material behavior for this hologram recording architecture, we decided to use a HPDLC photopolymer. The monomer used was dipentaerythritol penta-/hexaacrylate (DPHPA), with a refractive index *n* = 1.490. We used the nematic liquid crystal BL036 from Merck. It is a mixture of 4-cyanobiphenyls, with alkyl chains of different lengths. It has an ordinary refractive index *n*_0_ = 1.5270 and a difference between extraordinary and ordinary index Δ*n*_e_ = 0.2670 [31]. The liquid crystal concentration was set at 28 wt% as the starting point for component optimization, and remained practically unchanged during this process. N-vinyl-2-pyrrolidone (NVP) was used as crosslinker, N-phenylglicine (NPG) as radical generator, octanoic acid (OA) as cosolvent, and ethyl eosin (YEt) as dye. The prepolymer solution was made by mixing the components under a red light, to which the material is not sensitive, using the quantities presented in Table 1. The solution was sonicated in an ultrasonic bath, deposited between glass plates 2 mm thick, and separated using glass microspheres as spacers. The microspheres were provided by Whitehouse Scientific, with a thickness between 20 and 30 μm. We take 30 µL of solution for each hologram coupler fabrication. The solution is deposited on the glass substrate (20 cm × 4 cm), just on the corners, and is distributed placing the other substrate over the solution and making pression with tweezers. 

**Figure 7:**
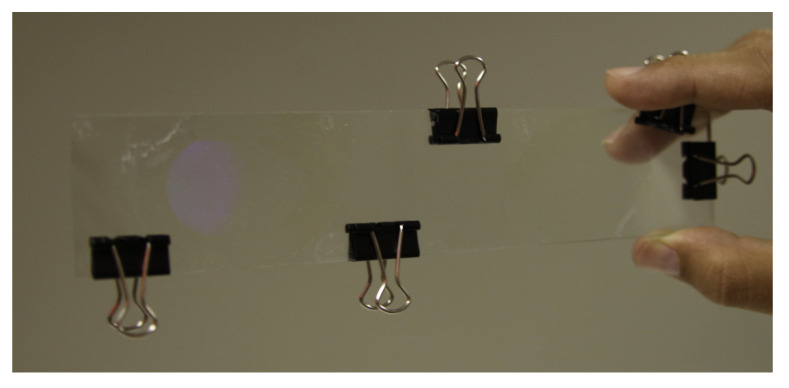
Photography of the recorded waveguide.

The experimental device is a typical transmission holographic setup. A Nd:YAG laser tuned at a wavelength of 532 nm was used to record diffraction gratings by means of continuous laser exposure. The laser beam was split into two secondary beams with an intensity ratio of 3:1, due to the low cross section of one of the beams. The normal section of the beams was increased to 3 cm^2^ using a spatial filter and collimating lens, while spatial filtering was ensured. The working intensity at 532 nm was 3 mW/cm^2^. Non-slanted diffraction gratings were recorded with two different spatial frequencies. We monitored the transmission efficiency, diffracted is trapped inside the wave guide, using red light (λ = 633 nm), which the dyes do not absorb. After recording, the sample was rotated to record the angular response around the first Bragg condition. In Figure 7, a photograph of the waveguide after recording is shown.

## 6. Results and Discussion

**Figure 8:**
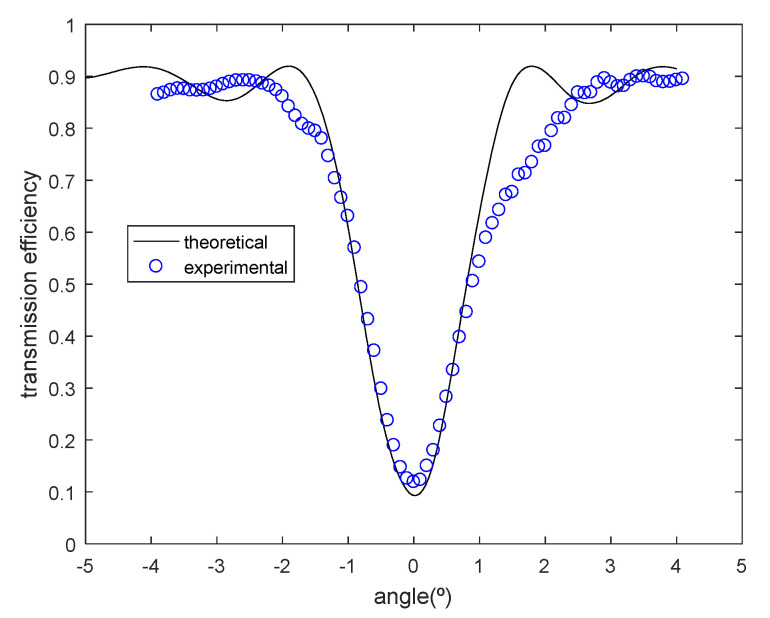
Transmission efficiency as a function of the angle made by the incident ray with respect to the normal of the sample for a slanted recorded grating of spatial frequency 1690 lines/mm.

Firstly, we will analyze two transmission gratings recorded by geometry in Figure 3. The angular response of the transmission efficiency was monitored as a function of the angle made by the incident ray with respect to the normal of the sample for slanted transmission diffraction gratings recorded in DPHPA photopolymer, following the configuration of Figure 3. In Figure 8, the experimental data and the theoretical fits using rigorous coupled-wave theory [30] are represented for a transmission grating. In this case, the grating has a spatial frequency of 1690 lines/mm, and the slant angle of the fringes with respect to the x axis is *ϕ* = 68.9°. After the fitting, the values for the theoretical model were a refractive index modulation of Δ*n* = 0.0107 ± 0.0001 and a thickness of d = 20 ± 1 μm. From Figure 8, it can be seen that although the maximum diffraction efficiency attainable is limited by reflection losses, and also, by absorption and scattering to near 92%, a diffraction efficiency of 80% was obtained in this case for the transmission gratings recorded in a DPHPA photopolymer. 

In order to get an idea of how much refractive index modulation is needed to obtain maximum diffraction efficiency, we used the following equation for the diffraction efficiency obtained by Kogelnik [32]

(19) $$\eta =sin^{2}\left(\frac{\pi \Delta nd}{\lambda \sqrt{c_{r}c_{s}}}\right)$$

where *c_r_* and *c_s_* are the cosine of the angles that the reference and object beam, respectively, form with the normal of the grating. The product Δ*nd* yielding to the maximum diffraction efficiency can be calculated as:

(20) $$\Delta nd=\frac{\lambda \sqrt{c_{r}c_{s}}}{2}$$

For the particular case of the transmission grating of Figure 8, this product must be Δ*nd* = 0.273 μm, whereas it was obtained a value of 0.214 μm. This means that there is still a 20% of field for improvement. 

Another important fact that can be observed from Figure 8 is that the Bragg condition is achieved at 0°, that is, at the required angle for achieving total internal reflection inside the glass, as explained in Section 2. The fact that the predicted Bragg angle coincides with the measured one also means that there is no shrinkage or swelling in this case.

**Figure 9:**
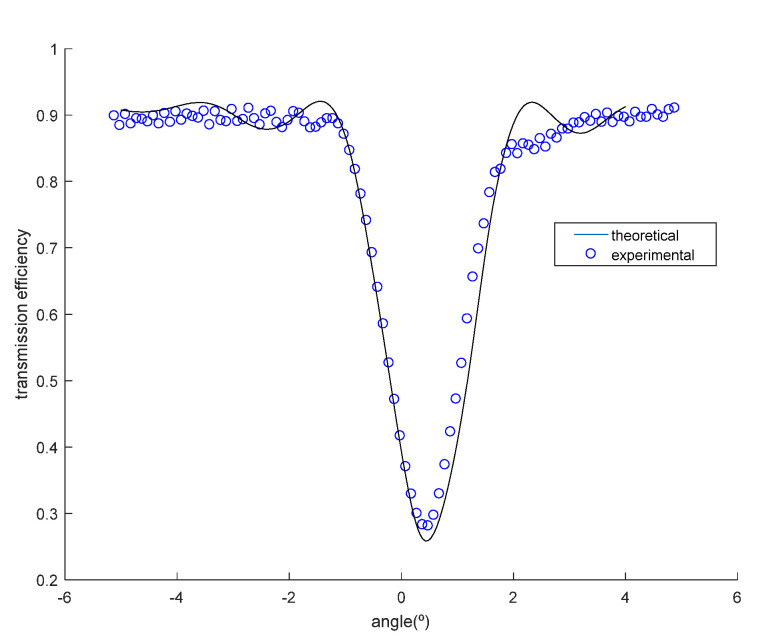
Transmission efficiency as a function of the angle made by the incident ray with respect to the normal of the sample for a slanted recorded grating of spatial frequency 1700 lines/mm.

The effects of shrinkage can be observed in Figure 9, where there is a small deviation of Bragg condition. The variation of the period and the slant angle after shrinkage, as explained in Section 4, alter the grating vector and therefore the Bragg angular condition. In this particular case, a factor of shrinkage of d’/d = 0.98 was calculated. The grating after shrinkage has 1700 lines/mm and a slant angle of the fringes of 68.5°. The fitted parameters obtained in this case were a refractive index modulation of Δ*n* = 0.0086 ± 0.0001 and a thickness of d = 20 ± 1 μm

**Figure 10:**
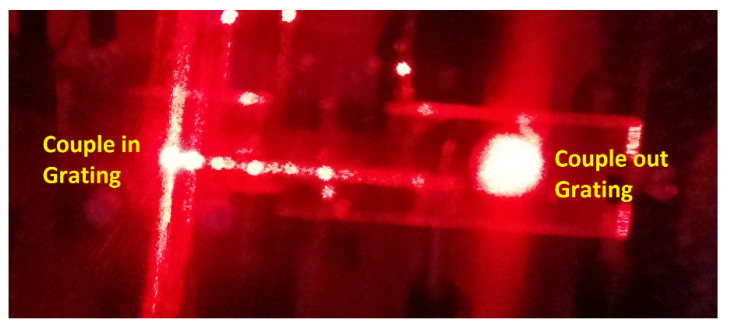
Recorded waveguide under illumination.

Once the material has proven to be useful for the recording of transmission gratings with the specifications of the couple in and couple out holograms explained in Section 2, a holographic waveguide was recorded with green light, and reconstructed with red light, as can be seen in Figure 10. Where the ability of the waveguide to redirect light from the couple in to the couple out hologram is evident. 

**Figure 11:**
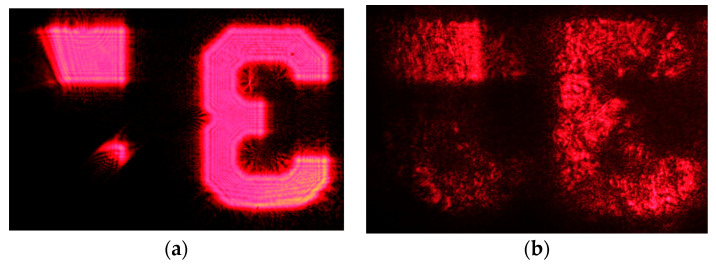
(**a**) Test image. (**b**) Image observed in the couple out hologram.

Finally, the device was tested as an imaging system so the couple in grating was illuminated by using a test image, and the final image was observed at the couple out hologram. Figure 11 shows the test image and also the image obtained. The grained structure of the image is basically due to speckle. On the other hand, it can be seen that another “3” with less intensity is formed to the left of the original one. This is due to the presence of a noise grating of the type presented in Figure 4b. As explained in Section 3, none of the other noise gratings of Figure 4 are “visible”, since the gratings recorded do not present swelling. The grating of Figure 4b is formed by the interference of the object beam with its reflected image. This noise grating has a spatial frequency 4370 lines/mm and a slant angle of 0°. The limiting shrinkage factor (d’/d) for this grating to disappear is (Equation (18)) 0.93, but it has been observed in the previous discussion that if there is shrinkage, this is very low, so this grating is present in the reconstruction process. Due to this reflection grating, some of the rays coming from the glass substrate are reflected, instead of being coupled by the couple out grating, and are extracted by the couple out grating in the other position, creating a second spurious image.

## 7. Conclusions

In this article, an optical see-through display based on a holographic waveguide has been designed and manufactured. Two transmission diffraction gratings of a spatial frequency of 1690 lines/mm were recorded onto a DPHPA polymer, acting as coupling gratings in the waveguide. The analysis of the angular response of the transmittance for these gratings demonstrate that an efficiency as high as 90% can be achieved in the material. It has also been studied the influence of the noise gratings in the imaging characteristics of the waveguide, showing that, in this case, the presence of a noise grating creates a secondary spurious image. 

## Figures and Tables

**Table 1:** Composition of the liquid solution for photopolymer holographic polymer dispersed liquid (HPDLC).

DPHPA (g)	BL036 (mL)	Yet (g)	NPG (g)	NVP (mL)	OA (mL)
1	0.590	0.001	0.03	0.330	0.090
